# Selective GPER activation decreases proliferation and activates apoptosis in tumor Leydig cells

**DOI:** 10.1038/cddis.2013.275

**Published:** 2013-08-01

**Authors:** A Chimento, I Casaburi, M Bartucci, M Patrizii, R Dattilo, P Avena, S Andò, V Pezzi, R Sirianni

**Affiliations:** 1 Department of Pharmacy, Health and Nutritional Sciences, University of Calabria, Arcavacata di Rende, Cosenza, Italy; 2 Department of Hematology, Oncology and Molecular Medicine, Istituto Superiore di Sanità, Rome, Italy

**Keywords:** apoptosis, Leydig cell tumor, GPER

## Abstract

We have previously shown that estrogens binding to estrogen receptor (ER) *α* increase proliferation of Leydig tumor cells. Estrogens can also bind to G protein-coupled ER (GPER) and activation of this receptor can either increase or decrease cell proliferation of several tumor types. The aim of this study was to investigate GPER expression in R2C rat tumor Leydig cells, evaluate effects of its activation on Leydig tumor cell proliferation and define the molecular mechanisms triggered in response to its activation. R2C cells express GPER and its activation, using the specific ligand G-1, is associated with decreased cell proliferation and initiation of apoptosis. Apoptosis after G-1 treatment was asserted by appearance of DNA condensation and fragmentation, decrease in Bcl-2 and increase in Bax expression, cytochrome c release, caspase and poly (ADP-ribose) polymerase-1 (PARP-1) activation. These effects were dependent on GPER activation because after silencing of the gene, using a specific small interfering RNA, cyt c release, PARP-1 activation and decrease in cell proliferation were abrogated. These events required a rapid, however, sustained extracellular regulated kinase 1/2 activation. G-1 was able to decrease the growth of R2C xenograft tumors in CD1 nude mice while increasing the number of apoptotic cells. In addition, *in vivo* administration of G-1 to male CD1 mice did not cause any alteration in testicular morphology, while cisplatin, the cytotoxic drug currently used for the therapy of Leydig tumors, severely damaged testicular structure, an event associated with infertility in cisplatin-treated patients. These observations indicate that GPER targeting for the therapy of Leydig cell tumor may represent a good alternative to cisplatin to preserve fertility in Leydig tumor patients.

Leydigioma is a rare testicular tumor that affects males at any age with two peaks of incidence, during prepuberty, between 5 and 10 years, and in adulthood between 25 and 35 years of age.^1^ The disease has a cure rate of 95%, however, compared with the general population, risk for second malignancies remains significantly increased for at least 35 years after treatment. Treatment for this type of cancer includes chemotherapy (especially alkylating agents) and radiotherapy. The testis has been shown to be highly susceptible to the toxic effects of irradiation and chemotherapy at all stages of life. The prepubertal testis is vulnerable because of its constant turnover of early germ cells and the maturation of the Leydig cell pool and other somatic compartments.^2^ Low-dose chemotherapy and/or radiotherapy can lead to temporary oligozoospermia (i.e., a sperm density in the ejaculate of <15 × 10^6^/ml) or azoospermia (i.e., no sperm in the ejaculate). If the damage is severe (e.g., as a result of high-dose treatment), all the spermatogonial stem cells commit to apoptosis or alternatively damaged Sertoli cells are unable to support the spermatogonial stem cells. This may lead to complete depletion of pool of spermatogonial stem cells and seminiferous tubules leading to Sertoli cells only pattern,^3^ and patient becomes permanently infertile. This event has been demonstrated also in mice administered with cisplatin at doses that reproduce those used in humans.^4^ Infertility will remain a significant adverse effect of testicular cancer therapy at all stages of life, therefore indications for a therapy with a reduced or transitory damage to the spermatogenetic process is deemed.

A large body of data indicate that estrogens regulate testis physiology,^5^ and are also involved in male gonadic diseases, including cancer.^6, 7^ Estrogen actions were thought to be exerted exclusively via nuclear estrogen receptors ER*α* (from ESR1 gene) and ER*β* (from ESR2 gene). Particularly, while nuclear ERs act as transcription factors to modulate activity of target genes by interacting with several DNA response elements,^8^ membrane-associated ERs elicit rapid non-genomic effects in response to estrogens.^9, 10, 11^ Estrogen actions through both ERs influence spermatogenesis in a cell-specific manner leading to germ cell proliferation, differentiation, as well as germ cell survival and apoptosis.^12^ ER*α* and ER*β* expression in the testis has been extensively studied, revealing their expression in rodent and human testicular normal,^13, 14^ and malignant cells.^15, 16^

We have previously shown that Leydig tumors produce estrogens that bind to ER*α* and activation of this receptor sustains cell proliferation.^16^ We also have shown that ERs antagonists such as hydroxytamoxifen and ICI182760 (ICI) are able to reduce proliferation of a rat Leydig tumor cell line.^16^ Similar effects were also found using letrozole, an aromatase (the enzyme that synthesizes estrogens from androgens) inhibitor. However, treatment of estrogen-dependent cancer with anti-estrogens frequently evolves in drug resistance.^17^ Another factor controlling tumor Leydig cell proliferation is IGF-I, whose production is increased in rat Leydig tumor cells,^16^ so the use of drugs targeting IGF receptor (IGF1R), blocking IGF-I effects, could also be suggested for the treatment of this type of tumor. Monoclonal antibodies anti-IGF1R are used for the therapy of different tumors.^18^ Early studies justify the investigation of IGF1R as a target for cancer therapy, however, a phase-III study with an anti-IGF1R antibody combined with erlotinib in advanced non-small-cell lung cancer was terminated recently for safety reasons and lack of efficiency (ClinicalTrials.gov Identifier: NCT00673049). Similarly, the anti-IGF1R monoclonal antibody, figitimumab, has been used in phase-I clinical trials for the treatment of refractory adrenocortical carcinoma. However, no objective responses were seen in the refractory adrenocortical cancer patients.^19^ Indeed a new treatment for Leydig cell tumors is deemed.

Recently a seven-transmembrane spanning receptor named GPER (G protein-coupled ER) was demonstrated to be capable of mediating estrogen actions.^10^ Expression of GPER in the testis has been studied only in very recent years. Expression of this receptor has been found in normal and tumor Sertoli and Leydig cells,^20, 21^ and GPER is overexpressed in germ cell tumors,^22^ and its activation promotes seminoma cell proliferation *in vitro*.^23^ However, currently there are no data on the effects produced on germ cell number by long-term GPER stimulation *in vivo*. Activation of GPER leads to the activation of downstream pathways that, depending on the cell type, are associated with both proliferation^24, 25^ and apoptosis.^26^ A very interesting study has highlighted the opposite effects played by GPER activation on cell proliferation of ER-negative and ER-positive breast cancer cells.^27^ Specifically, when ERs are expressed, the activation of GPER leads to inhibition of cell proliferation. On the contrary, when cells are ER negative, activation of GPER leads to an increase in cell proliferation. Tumor Leydig cells express all three ERs; however, the effect of GPER-selective activation on the proliferation of tumor Leydig cells is unknown.

The aim of this study was to investigate both *in vitro* and *in vivo* effects of GPER-selective agonist G-1 on R2C rat tumor Leydig cell growth. In addition, we wanted to evaluate the effects produced by GPER-selective activation on testis morphology *in vivo*. Results from this study indicate GPER as a new target for the therapy of Leydig tumors, without affecting testicular function.

## Results

### GPER is expressed in R2C rat tumor Leydig cells

**Figure 1:**
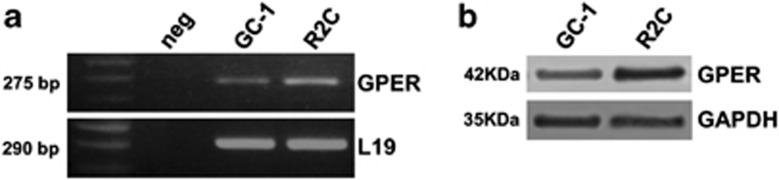
Expression of GPER in R2C cells. (**a**) GPER mRNA expression in R2C cells was analyzed by RT-PCR; GC-1 cells were used as positive control; negative control (neg) contained water instead of cDNA. L19 was used as housekeeping gene. Size in base pair of amplified fragments is indicated. (**b**) Western blot analysis of GPER was performed on 50 *μ*g of total proteins extracted from GC-1 and R2C cells. GAPDH was used as a loading control. Blots are representative of three independent experiments with similar results

We first investigated GPER expression in R2C cells, a valid model for Leydigioma. GPER mRNA and protein are expressed in R2C cells, and expression levels are comparable to GC-1, an immortalized mouse spermatogonial cell line that we used as positive control (Figures 1 and b).^28^

### E2 and G-1 exert opposite effects on R2C cell growth

**Figure 2:**
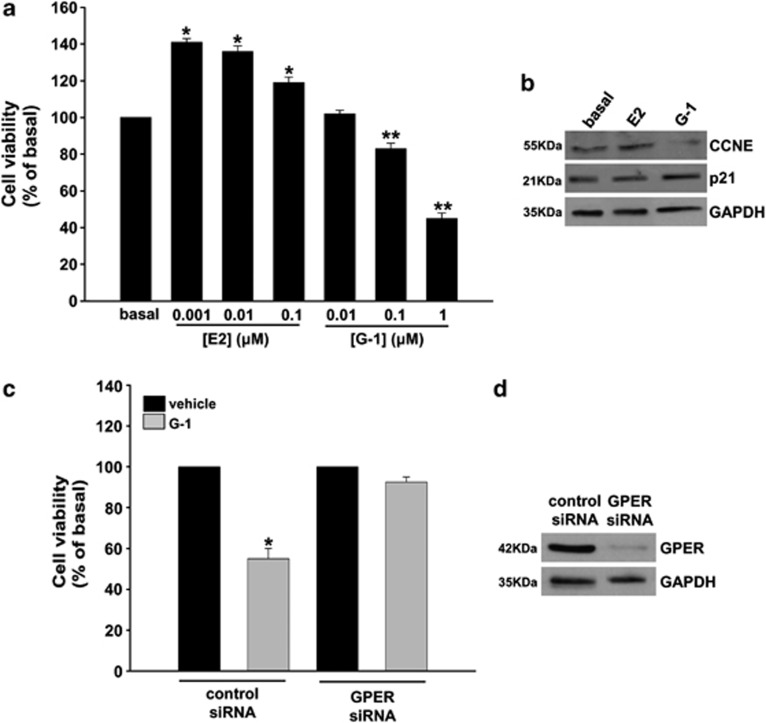
Effects of GPER-selective activation on R2C cell proliferation. (**a**) R2C cells were treated with increasing concentrations of E2 or G-1 for 72 h. Cell proliferation was evaluated by MTT assay. Results were expressed as mean+S.E. of three independent experiments each performed in triplicate. Statistically significant differences are indicated (*P<0.01 and **P<0.05 compared with basal). (**b**) R2C cells were treated with E2 (1 nM) and G-1 (1 *μ*M) for 48 h. Western blot analyses of cyclin E (CCNE) and p21 were performed on 50 *μ*g of total proteins. Blots are representative of three independent experiments with similar results. GAPDH was used as a loading control. (**c**) R2C cells were transfected with GPER or non-targeting (control siRNA) siRNA as indicated. Twenty-four hours after transfection, cells were treated for an additional 48 h with G-1 (1 *μ*M). Proliferation was evaluated by MTT assay. Results were expressed as mean+S.E. of three independent experiments each performed in triplicate. (**d**) R2C cells were transfected with GPER or non-targeting (control siRNA) siRNA as indicate. Seventy-two hours after transfection, cells were lysed and subject to western blot analyses for GPER. GAPDH was used as a loading control. Results are representative of at least three independent experiments

R2C cells express ER *α* and *β* and treatment of these cells with 17*β*-estradiol (E2) increases cell proliferation.^16^ Starting from these observations, we decided to examine the effects of G-1, a selective GPER ligand, on R2C cell proliferation. Cells were treated for 72 h with E2 and G-1. While E2 caused an increase in cell proliferation, G-1 produced a dose-dependent reduction in R2C cell growth (Figure 2a). To define the mechanism underlying the opposite effects elicited by E2 and G-1 on cell growth, we analyzed the expression of cyclin E, a well-known estrogen-dependent cell cycle regulator.^29^ As previously shown,^16^ a significant increase in cyclin E expression levels was observed after 48 h treatment with E2, while the presence of G-1 produced opposite effects decreasing cyclin E protein content compared with cells treated with vehicle alone (Figure 2b). To explain if this event was associated with a cell cycle arrest, we investigated expression of G1 phase marker, such as cyclin-dependent kinase inhibitor WAF1/p21 (p21). While treatment with E2 did not determine any change in p21 expression, the use of G-1 increased p21 protein levels (Figure 2b) compared with untreated cells.

In order to confirm that GPER is required to produce G-1-dependent decrease in R2C cell proliferation, we decided to knockdown its expression by using gene silencing technology. The presence of a specific small interfering RNA (siRNA) for GPER was able to abrogate the inhibitory effects exerted by G-1 on R2C cell proliferation (Figure 2c). GPER gene silencing was assessed by western blot analysis (Figure 2d).

### G-1 causes DNA damage consequent to a mitochondria-dependent apoptotic pathway

**Figure 3:**
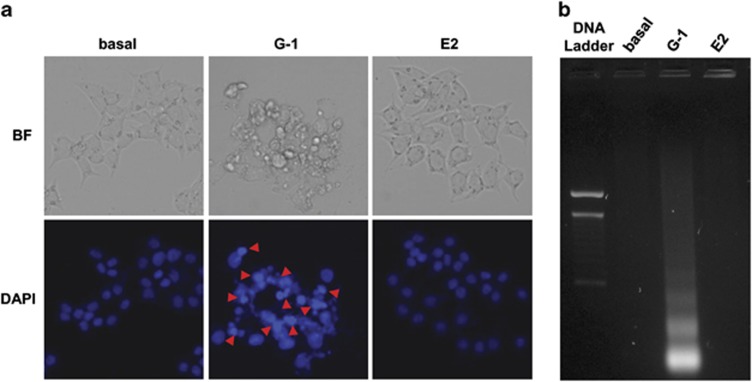
Effects of GPER activation on R2C nuclei morphology. (**a** and **b**) R2C cells were left untreated (basal) or treated with G-1 (1 *μ*M) and E2 (1 nM) for 72 h. (**a**) After treatment, R2C cells were fixed with paraformaldehyde, dyed with DAPI and observed under fluorescent microscope (magnification × 400). Arrows indicate condensed nuclei. Images are from a representative experiment. (**b**) After treatment, DNA was extracted from cells and analyzed on a 1.5% agarose gel. Images are from a representative experiment

We then wanted to verify if G-1-dependent decrease in cell growth was correlated with apoptosis. 2-(4-Amidinophenyl)-6-indolecarbamidine dihydrochloride (DAPI) staining demonstrated that untreated R2C cells had round nuclei with regular contours and large in size. After 72-h treatment with G-1, cells showed shrunken and irregularly shaped or degraded nuclei with condensed DNA, events not observed after exposure to E2 for the same period of time (Figure 3a). Chromatin condensation was associated with DNA fragmentation only with G-1 but not E2. In fact, gel electrophoresis of DNA extracted from R2C cells after 72 h treatment demonstrated the classic laddering pattern of inter-nucleosomal DNA fragmentation that was absent in control and E2-treated cells (Figure 3b). Evaluation of G-1 effect on Fas and FasL mRNA expression, known markers for the extrinsic apoptotic pathway, demonstrated no effect of GPER activation (data not shown).

**Figure 4:**
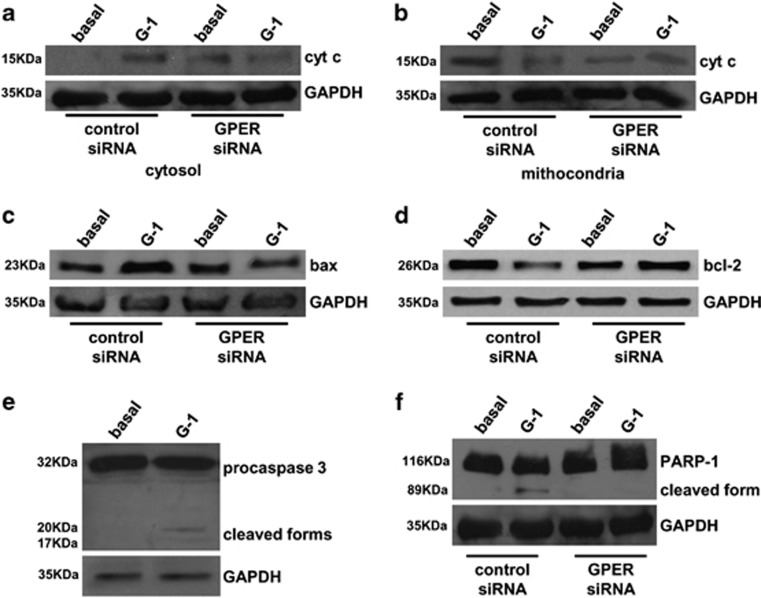
G-1 activates a mitochondria-dependent apoptotic pathway. (**a**-**d**) R2C cells were transfected with GPER non-targeting (control siRNA) siRNA (50 nM) as indicated. Twenty-four hours after transfection, cells were treated for an additional 24 h with G-1 (1 *μ*M). Cytochrome c (cyt c) in cytosolic (**a**) and mitochondrial (**b**) fractions, bax (**c**) and bcl-2 (**d**) levels were detected by western blot analysis. GAPDH was used a loading control. Blots are representative of three independent experiments with similar results. (**e**) R2C cells were treated with G-1 (1 *μ*M) for 24 h. Western blot analyses of caspase 3 cleaved forms were determined by western blot analysis on 50 *μ*g of total proteins. Blots are representative of three independent experiments with similar results. GAPDH was used as a loading control. (**f**) R2C cells were transfected with GPER and non-targeting (control siRNA) siRNA (50 nM) as indicated. Twenty-four hours after transfection, cells were treated for an additional 24 h with G-1 (1 *μ*M). Fifty micrograms of total proteins were analyzed by western blot for PARP-1 activation. GAPDH was used as a loading control. Blots are representative of at least three independent experiments with similar results

When intrinsic apoptotic mechanism is stimulated, cytochrome c is released from the mitochondria into the cytosol.^30^ Cytosolic translocation of cytochrome c has been proposed to be an essential component in the mitochondria-dependent apoptotic pathway. Therefore, we first examined cytochrome c release into the cytosol after GPER activation. Cell lysates were fractionated into cytosolic and mitochondrial fractions and were analyzed by western blot analysis (Figures 4 and b). Cytochrome c levels increased in the cytosolic fraction of treated samples (Figure 4a) and instead decreased in the mitochondrial one (Figure 4b). Importantly, GPER silencing completely blocked G-1 effect, further highlighting the specific involvement of the G protein-coupled receptor in the G-1-dependent effect (Figures 4a and b). As bcl-2 family members have pivotal roles in regulating the mitochondrial apoptotic pathway, bax and bcl-2 protein levels were evaluated by western blot analysis. The presence of G-1 decreased bcl-2, while increased bax expression (Figures 4c and d). Importantly, when GPER was silenced with a selective siRNA, the G-1-dependent increase in bax (Figure 4c) and decrease in bcl-2 (Figure 4d) expression were lost. Cytochrome c release triggers caspase activation,^31^ for this reason we examined the activation of a critical executor of cell apoptosis, caspase 3, by western blot analysis. After 24-h treatment with G-1, caspase 3 cleaved forms were visible (Figure 4e). Once activated caspase 3 leads to the cleavage and inactivation of poly (ADP-ribose) polymerase-1 (PARP-1), involved in the regulation of DNA repair.^32^ PARP-1 was inactivated, as seen by the presence of cleaved forms, after 24 h of treatment with G-1 and its cleavage was lost after GPER silencing (Figure 4f).

### G-1 induces sustained ERK1/2 activation

**Figure 5:**
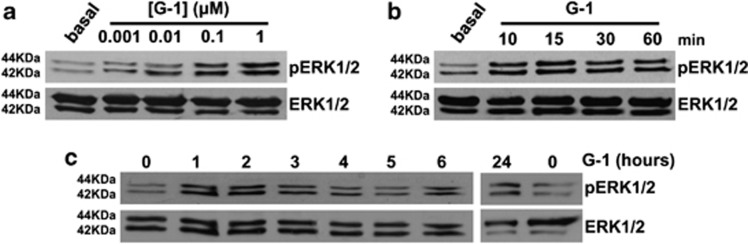
Effects of G-1 on ERK1/2 activation. (**a**) R2C cells were treated for 10 min with the indicated concentrations of G-1. Western blot analysis of pERK1/2 was performed on 20 *μ*g of total proteins. ERK1/2 were used as a loading control. Blots are representative of three independent experiments with similar results. (**b** and **c**) R2C cells were treated for the indicated times with G-1 (1 *μ*M). Western blot analysis of pERK1/2 was performed on 20 *μ*g of total proteins. ERK1/2 were used as a loading control. Blots are representative of at least three independent experiments with similar results

In order to define the molecular mechanism associated with G-1-dependent apoptosis, we investigated activation of MAPK family members extracellular signal-regulated kinase 1/2 (ERK1/2), which have been demonstrated to be involved in apoptosis if activated for a prolonged time.^33^ As shown in Figure 5a, ERK1/2 were activated by G-1 treatment, as seen by increased levels of phosphorylation, in a dose-response manner, with 1 *μ*M being the most effective dose. Activation was already visible after 10-min treatment with G-1 (Figure 5b) and persisted for up to 6 h (Figure 5c). Investigation of G-1 effect on ERK1/2 activation at later times (24 h) demonstrated a higher phosphorylated status compared with untreated cells (Figure 5c).

### *In vivo* evaluation of G-1- and cisplatin-mediated effects on xenograft tumor growth and testis histopathology

**Figure 6:**
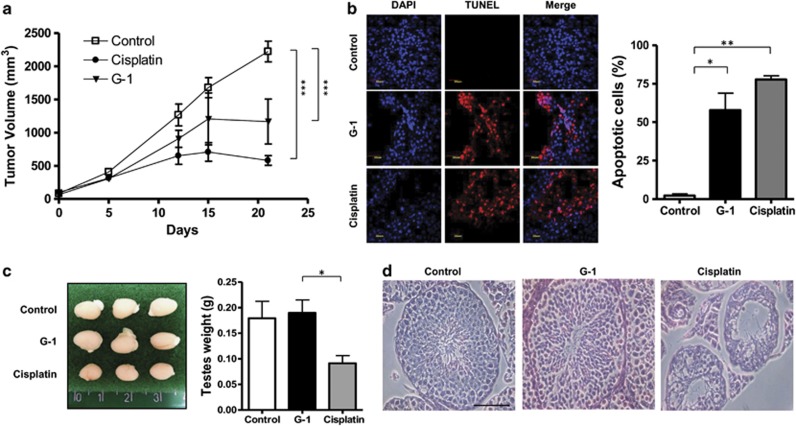
G-1 reduces Leydig tumor xenograft growth and increases apoptosis *in vivo* without affecting testicular structure. (**a**) R2C cells were injected in male nude mice and tumors were allowed to grow to the size of ∼200 mm^3^ before mice were assigned randomly in three groups of eight: group 1 received 0.1 ml PBS IP only as control; Group 2 and Group 3 received for 20 days, every other day, 2.5 mg/kg of cisplatin or 1 mg/kg of G-1, respectively. Tumor volumes (mm^3^) were measured regularly throughout the study. Data are represented as the mean of eight tumors from each group. (**b**) Representative TUNEL (red) and DAPI (blue) double staining acquired with a × 60 objective on R2C xenografts sections at day 22. Fluorescence was photographed using an Olympus FV-1000 spectral confocal microscope equipped with an UltraPlan Apochromatic 60X N.A.1.35 objective Right panel shows the percentage of TUNEL-positive cells *versus* DAPI in R2C xenografts. (**c**) Left panel: representative picture indicating the difference in testicular weight under the different treatments. Right panel: average testicles weight from untreated (control) G-1- and cisplatin-treated mice (*n*=8 per each condition). (**d**) Representative H&E performed on paraffin-embedded testis sections 2 days after treatment withdrawal (day 22). Photomicrograph of cross-sections of the testes of control, cisplatin and G-1-treated mice. In seminiferous tubules with normal aspect (control and G-1), germ cells are organized in concentric layers and the tubular lumen is empty; samples from mice treated with cisplatin showed altered seminiferous tubules with irregular shape, epithelial disorganization and detached germ cells (H&E staining, × 400 bar: 50 μm). A *P*<0.05 is represented by a single asterisk, a *P*<0.01 is represented by a double asterisk, while three asterisks indicate *P*<0.001

Based on *in vitro* results showing that GPER activation by G-1 exerts anti-proliferative and apoptotic effects on R2C cells, we established R2C xenograft tumors in male immunocompromised mice to investigate the ability of G-1 to control tumor growth *in vivo.* We also wanted to compare the effects mediated by G-1 with those exerted by cisplatin, an agent commonly used for the treatment of testicular cancer that while controlling tumor growth damages the spermatogenic process causing infertility.^34^ R2C cells were injected into the intrascapular region of male nude mice, when the tumor reached an average volume of 0.2 cm^3^, animals were randomized into three groups to be treated with either vehicle, G-1 or cisplatin. As shown in Figure 6a, both G-1 and cisplatin caused a significative regression in tumor growth, with cisplatin more effective than G-1. Both treatments were able to induce apoptosis, as evidenced by the presence of terminal deoxynucleotidyl transferase-mediated deoxyuridine triphosphate nick-end labeling (TUNEL)-positive nuclei (Figure 6b). However, evaluation of testicular weight showed a significant reduction in cisplatin-treated mice compared with normal and G-1-treated animals (Figure 6c). Examination of testicular histopathology of cisplatin-exposed testis cross-sections revealed severe atrophy and germ cell loss, decreased cellularity and a reduction in the height of the seminiferous epithelium (Figure 6d). In addition, apical sloughing, shedding of cellular material and absence of specific cell populations were seen in as many as 90% of tubules in a given testis cross-section. By contrast, the evaluation of hystopathological features of seminiferous tubules from G-1-treated mice showed a normal cellularity and morphology, very similar to those of the control group (Figure 6d).

These results show a significant inhibitory effect on tumor growth by G-1 without the side effects of cisplatin on the structural and functional elements of the testis.

## Discussion

In this study, we have shown that GPER is a good target to reduce Leydig tumor proliferation. In fact, GPER is expressed in this type of cancer and its activation is associated with a drastic reduction of cell proliferation consequently to the initiation of mitochondria-dependent apoptotic pathway. This mechanism has been demonstrated for other tumor cell types including those of the breast^27^ and prostate.^26^

The literature of the recent years has better defined the different roles played by the different ERs (ER*α*, ER*β* and GPER) in normal and malignant cell proliferation. In cancer cells, when all three ERs are expressed, it appears that the major proliferative effects are exerted by ER*α*^27^ while activation of ER*β* and GPER is linked to apoptosis.^26, 27, 35^ On the contrary, when ER*α* is absent GPER is the receptor-activating cell proliferation.^36, 37^

In agreement with this hypothesis of estrogens mediating proliferative effects through a different receptor type based on their relative expression, in our model of Leydig cell tumor expressing all three receptors, we have previously demonstrated that ER*α* mediates proliferation^16^ and here we showed that GPER activation triggers apoptosis.

It could be asked why ER*α*-dependent proliferative effects prevail on GPER-dependent inhibitory effects. It could be explained in view of the slightly different kD that E2 has for the two receptors, 0.055 nM for ER*α*^38^ and 3 nM for GPER,^39^ and also considering the relative expression levels of the two receptors with eventually ER*α* present at higher levels than GPER.

It has been demonstrated that GPER is overexpressed in human seminomas, compared with normal testis, and in a seminoma cell line in comparison with a normal spermatogonial cell line.^22^ Expression of ER*α* and ER*β* in germ cell tumors has been evaluated and compared with normal testis.^40^ ER*β* expression was found in the majority of tumor cells, however, at lower levels when compared with expression in germ cells of the normal testis. On the other hand, ER*α* was not expressed in any seminoma, endodermal sinus tumors, embryonal carcinoma, mature teratoma or mixed germ cell tumors, suggesting that in these types of tumors estrogens exert proliferative effects through a different receptor. Other independent researchers showed that seminomas and embryonal carcinomas had a positive ER*β*1 and ER*β*2 immunoreactivity, while ER*α* signal was undetectable.^41^ Indeed in a seminoma cell line, which lacks of ER*α* expression, ER*β* activation has been shown associated with cell necrosis and autophagy.^42^ Opposite to this effect, GPER activation using the specific ligand G-1 increased proliferation of a human testicular seminoma cell line.^23^ Collectively, these data further support the hypothesis of a mechanism that sees the involvement of a specific type of ER in cell growth based on the relative expression of the ERs.

Apoptosis can be induced by the extrinsic^43^ and intrinsic^44^ mechanisms. Our data clearly indicate that GPER activation by the specific ligand G-1 is associated with the initiation of the intrinsic apoptotic mechanism. We demonstrated the induction of apoptosis through DAPI staining, which evidenced nuclei morphological changes and a laddering pattern of inter-nucleosomal DNA. Bcl-2 family of proteins has a central role in the intrinsic apoptotic mechanism.^45^ This family consists of both pro- (bax, bad, bak and bid) and anti-apoptotic (bcl-2 and bcl-xl) proteins that modulate the execution phase of the cell death pathway. Bax exerts pro-apoptotic activity by allowing cytochrome c translocation from the mitochondria to cytosol,^46^ where it binds to apoptotic protease-activating factor-1,^47^ which in turn binds to procaspase 9 resulting in its activation,^48^ responsible for the proteolytic activation of executioner caspase 3.^49^ The active caspase 3 is then involved in the cleavage of a set of proteins including PARP-1.^32^ Bcl-2, instead, exerts its anti-apoptotic activity, at least in part, by inhibiting the translocation of bax to the mitochondria.^47^ All these events were observed in Leydig tumor cells in response to GPER activation. Silencing of the GPER gene confirmed the role of this receptor in the activation of a mitochondria-dependent apoptotic pathway. In fact, the reduced expression of GPER completely abolished the effects of G-1 on cytochrome c release in the cytosol, on the increase in bax or the decrease in bcl2 expression and on PARP-1 inactivation. These events require a rapid, however sustained, activation of the MAPK family members ERK1/2. Our data are in agreement with previous reports demonstrating that transient activation of ERK1/2 has a pivotal role in cell proliferation and that sustained ERK1/2 activation induces cell cycle arrest^50^ and death.^33^ The ability of G-1 to reduce the growth of R2C *in vitro* was also evaluated *in vivo*. G-1 significatively inhibited the growth of R2C xenografts and increased the number of apoptotic cells.

One could question if the use of G-1 for the therapy of Leydig tumors could indeed affect normal spermatogenesis. Our *in vivo* experiments demonstrated that administration of G-1 for >2 weeks period did not cause any damage to the normal testis structure, opposite to what seen with cisplatin.

We have investigated expression of GPER in mouse and rat germ cells at differential maturative stages.^28, 51, 52, 53^ Importantly, GPER activation induces proliferation of spermatogonia,^28^ and can regulate the physiological process of apoptosis of pachytene spermatocytes,^51, 53^ and round spermatids during spermatogenesis.^52^ Spermatogonia represent the stem cells of male germ cells, so it could be speculated that the use of a GPER-specific agonist for the therapy of Leydig tumors would not affect normal spermatogenesis allowing preservation of fertility in patients treated for this type of tumor. On the other hand, chemotherapeutic agents currently used for the treatment of testicular cancers, such as cisplatin, despite their potent anti-neoplastic action, have several side effects including nephrotoxicity,^54^ peripheral neuropathy^55^ and azoospermia.^56^ This last event is dependent on a reduction in the number of spermatogonia, which appear to be the most sensitive germ cell type to cisplatin.^57^

In conclusion, although further studies are needed, our results point out how GPER and its agonists such as G-1 can be considered as a potential new pharmacological tool to reduce the growth of Leydig cell tumors. This drug, opposite to the current used drug, does not seem to affect germ cells viability and thus could preserve male fertility.

## Materials and Methods

### Cell cultures

Cells were obtained from ATCC (LGC Standards, Teddington, Middlesex, UK), grown for 2 weeks (four passages) before freezing aliquots. Each aliquot was used for no >10 passages. R2C cells (a rat Leydig tumor cell line) were cultured in Ham/F-10 medium supplemented with 15% horse serum, 2.5% fetal bovine serum (FBS) and 1% penicillin/streptomycin (Sigma-Aldrich, Milano, Italy; complete medium).^58^ For experiments, cells were plated in complete medium, 48 h later treated in Ham/F-10 with antibiotics and without serum (serum-free medium) for the indicated times, G-1 (Tocris Bioscience, Ellisville, MO, USA) and E2 (Sigma-Aldrich) at the indicated concentrations. GC-1 cells, a mouse spermatogonia type B cell line (ATCC), were cultured in DMEM/F-12 medium supplemented with 10% FBS, 1% glutamine and 1% penicillin/streptomycin (Sigma-Aldrich).

### RNA extraction and RT-PCR

TRizol RNA isolation system (Invitrogen, Carlsbad, CA, USA) was used to extract total RNA from GC-1 and R2C cells. Each RNA sample was treated with DNase I (Ambion, Austin, TX, USA), and purity and integrity of the RNA were confirmed spectroscopically and by gel electrophoresis. One microgram of total RNA was reverse transcribed in a final volume of 30 *μ*l using the ImProm-II reverse transcriptase system kit (Promega Italia SRL, Milano, Italy). cDNAs were used for PCR. PCR amplification was performed using 1.5 U of Taq DNA polymerase (Promega Italia SRL) in PCR buffer containing 200 *μ*M dNTP, 1.5 mM MgCl_2_ and 25 pmoles of each primer in a total volume of 50 *μ*l. GPER PCR was performed as previously described.^51^ L19 ribosomal protein mRNA was used as housekeeping gene. PCR products were analyzed by electrophoresis on a 2% agarose gel stained with ethidium bromide (Sigma-Aldrich).

### Western blot analysis

Fifty micrograms of protein was subjected to western blot.^16^ Blots were incubated overnight at 4 °C with specific antibodies: anti-GPER (MBL International Corporation, Woburn, MA, USA), anti-cyclin E, anti-p21, anti-cytochrome c, anti-bax, anti-bcl-2, anti-caspase 3, anti-Parp-1 and anti-glyceraldehyde 3-phosphate dehydrogenase (GAPDH; Santa Cruz Biotechnology, Santa Cruz, CA, USA), anti-phosphoERK-42/44 and anti-ERK-42/44 (Cell Signaling Technology, Beverly, MA, USA). Membranes were incubated with horseradish peroxidase-conjugated secondary antibodies (Amersham Pharmacia Biotech, Piscataway, NJ, USA) and immunoreactive bands were visualized by ECL (Amersham Pharmacia Biotech).

### Cytochrome c detection

Cells were cultured in complete medium for 48 h in 100 mm dishes (7 × 10^6^ cells), then treated in serum-free medium for 24 h as previously reported.^53^ Cytochrome c was detected by western blot analysis in mitochondrial and cytoplasmic fractions. Cells were harvested by centrifugation at 2500 r.p.m. for 10 min at 4 °C. Pellets were resuspended in 50 *μ*l of sucrose buffer (250 mM sucrose; 10 mM Hepes; 10 mM KCl; 1.5 mM MgCl2; 1 mM EDTA; 1 mM EGTA; Sigma-Aldrich) containing 20 *μ*g/ml aprotinin, 20 *μ*g/ml leupeptin, 1 mM PMSF and 0.05% digitonine (Sigma-Aldrich). Cells were incubated for 20 min at 4 °C and then centrifuged at 13 000 r.p.m. for 15 min at 4 °C. The supernatant containing cytosolic protein fraction was transferred to new tubes and the resulting mitochondrial pellet was resuspended in 50 *μ*l of lysis buffer (1% Triton X-100; 1 mM EDTA; 1 mM EGTA; 10 mM Tris–HCl, pH 7.4; Sigma-Aldrich) containing 20 *μ*g/ml aprotinin, 20 *μ*g/ml leupeptin, 1 mM PMSF and then centrifuged at 13 000 r.p.m. for 10 min at 4 °C. Equal amounts of proteins were resolved by 11% SDS/polyacrylamide gel as indicated in the western blot analysis paragraph.

### RNA interference

The Stealth RNAi Negative Control (low GC content) and siRNA oligoribonucleotide duplex to rat GPER (5′-ACGCUCAAGGCAGUCAUACCAGACA-3′) were purchased from Invitrogen. Cells were plated into 60 mm dishes, at 4 × 10^6^ cells, for protein extraction, and into 24-well plates, at 2 × 10^6^ cells, for proliferation assay and used for transfection 48 h later. siRNAs were transfected to a final concentration of 50 nM using the Lipofectamine 2000 reagent, used according to the manufacturer's recommendations (Invitrogen). GPER-specific knockdown was checked by western analysis of proteins extracted from cells transfected for 48 h and then treated for 24 h. Proliferation was evaluated for cells transfected for 24 h and then treated for 48 h.

### Determination of nuclear morphological changes

Cells were cultured in complete medium for 48 h on microscope slides (1 × 10^5^ cells), then treated in serum-free medium for 72 h. Cells were washed with PBS and fixed in 4% formaldehyde for 10 min at room temperature. Fixed cells were washed with PBS and incubated with DAPI (0.2 *μ*g/ml; Sigma-Aldrich) for 10 min in a humidified chamber, protected from light, at 37 °C. Cells were then washed three times with cold PBS and one drop of mounting solution was added. Cell nuclei were observed and imaged by an inverted fluorescence microscope ( × 400 magnification) with excitation at 350 nm and emission at 460 nm. The number of apoptotic nuclei was determined in at least six randomly selected areas from three cover slips of each experimental group.

### Determination of DNA fragmentation

Cells were cultured in complete medium in 100 mm dishes (7 × 10^6^ cells) for 48 h, and then treated in serum-free medium for 72 h. To determine the occurrence of DNA fragmentation, total DNA was extracted from control and G-1 (1 *μ*M) treated cells as previously described.^53^ Equal amounts of DNA were analyzed by electrophoresis on a 2% agarose gel stained with ethidium bromide (Sigma-Aldrich).

### Assessment of cell proliferation

The effect of E2 and G-1 on cell viability was measured using 3-[4,5-dimethylthiaoly]-2,5-diphenyltetrazolium bromide (MTT) assay as previously described.^59^ Briefly, cells were cultured in complete medium in 24-well plates (2 × 10^5^ cells per well) for 48 h, and then treated in serum-free medium for 72 h. Seventy-two hours after treatment, fresh MTT (Sigma-Aldrich), re-suspended in PBS, was added to each well (final concentration 0.33 mg/ml). After 30-min incubation, cells were lysed with 1 ml of DMSO (Sigma-Aldrich). Each experiment was performed in triplicate and the optical density was measured at 570 nm in a spectrophotometer.

### *In vivo* studies

Six-week-old male CD1 nude mice from Charles River Laboratories (Wilmington, MA, USA) were maintained in accordance with the institutional guidelines of the Istituto Superiore di Sanità and all the procedures were approved by local Ethics Committee for Animal Research. Mice were housed in group of four in standard rodent cages and food and water were provided *ad libitum*. To obtain synchronized tumors, 150 000 R2C cells were injected subcutaneously into the right flank of each mouse. Tumors were allowed to grow to the size of ∼0.2 cm^3^ before the administration of compounds. For experimental procedures, mice were divided in three groups:

In group 1 (*n*=8), used as control, mice received intraperitoneal injections (IP)  of 0.1 ml PBS.

In group 2 (*n*=8), mice were treated IP with 2.5 mg/kg cisplatin (Sigma Aldrich).

In group 3 (*n*=8), mice were administered IP with 1 mg/kg G-1  (Tocris Bioscience, Bristol UK).

In all three groups, administrations were carried out every other day and for 20 consecutive days. Tumor growth was evaluated with an electronic caliper before every administration. Two days post treatments termination all mice were killed by cervical dislocation. Tumors and testes were removed and weighed using a PL202-L Precision Balance (Mettler-Toledo, Novate Milanese, Italy). Immunofluorescence and histological analysis were performed on frozen or formalin fixed/paraffin-embedded tissues. H&E staining was performed on 5 *μ*m paraffin-embedded testis sections and observed through a Nikon Eclipse E1000 transmitted light right microscope equipped with PlanFluor 20 × dry objective (Nikon, Melville, NY, USA). Images were subsequently taken by using a Nikon DXM1200 RGB camera and the Nikon ACT-1 software. To assess G-1 *versus* cisplatin efficacy, 5 *μ*m thick frozen xenografts sections were cut and incubated with TUNEL reaction mixture (Roche, Basel, Switzerland) for 1 h at RT. To evaluate the percentage of TUNEL-positive cells in tumor xenografts, image analysis was performed with ImageJ (http://rsb.info.nih.gov/ij/). Single channels were extracted from the confocal images either for nuclei (DAPI) or TUNEL (TMR-Red) and after application of a threshold that eliminates background dust, a watershed filter was applied on the binary images. The tool for particle analysis was used to quantify the amount of TUNEL positivity as compared with the number of DAPI-stained nuclei/particles.

### Histopathological analysis of testis sections

For evaluation of testicular histopathology, formaldehyde-fixed paraffin-embedded testis cross-sections (5 *μ*m) were transferred onto poly-ℒ-lysine-coated slides and stained with hematoxylin and eosin and examined under an Olympus light microscope (Olympus Italia Srl, Segrate Milano, Italy).

### Data analysis and statistical methods

All experiments were conducted at least three times and the results were from representative experiments. Data were expressed as mean values+S.E., and the statistical significance between control (basal) and treated samples was analyzed with SPSS10.0 statistical software (Systat Software Inc., London, UK). The unpaired Student's *t*-test was used to compare two groups. *P*<0.05 was considered statistically significant.

For *in vivo* studies, statistical analyses were performed using GraphPad Prism 4 (GraphPad Software Inc., www.graphpad.com). Data are presented as mean+S.E. Statistical significance was determined by ANOVA (one-way or two-way) with Bonferroni *post test*. A *P*-value <0.05 is represented by a single asterisk, a *P-*value <0.01 is represented by a double asterisk, while three asterisks indicate *P*<0.001.

## Abbreviations

DAPI: 2-(4-amidinophenyl)-6-indolecarbamidine dihydrochloride
E2: 17*β*-estradiol
ER: estrogen receptor
ERK1/2: extracellular regulated kinase 1/2
GAPDH: glyceraldehyde 3-phosphate dehydrogenase
GPER: G protein-coupled estrogen receptor 1
MTT: 3-[4,5-dimethylthiaoly]-2,5-diphenyltetrazolium bromide
PARP-1: poly (ADP-ribose) polymerase-1
siRNA: small interfering RNA
